# Rapamycin blocks the IL-13-induced deficiency of Epidermal Barrier Related Proteins via upregulation of miR-143 in HaCaT Keratinocytes

**DOI:** 10.7150/ijms.45765

**Published:** 2020-07-25

**Authors:** Qian-Nan Jia, Yue-Ping Zeng

**Affiliations:** 1 Department of Dermatology, Peking Union Medical College Hospital, Chinese Academy of Medical Sciences and Peking Union Medical College.; 2 National Clinical Research Center for Dermatologic and Immunologic diseases, Beijing, China.

**Keywords:** mTOR, atopic dermatitis, rapamycin, microRNA-143, IL-13

## Abstract

Interleukin (IL)-13 plays a key role in the pathogenesis of atopic dermatitis (AD). Our preliminary study demonstrated that forced expression of miR-143 could block IL-13-induced down-regulation of epidermal barrier related proteins in epidermal keratinocytes. As previous studies suggested that miR-143 expression was regulated by mammalian target of rapamycin (mTOR) signaling pathway, we investigated the mechanism of mTOR signaling pathway in the epidermal barrier dysfunction of AD. The HaCaT cells were stimulated by IL-13 and subsequently treated with rapamycin. The expression levels of miR-143, IL-13 receptor α1 (IL-13Rα1), p-mTOR, p-S6K1, p-Akt, and epidermal barrier related proteins were analyzed through RT-qPCR and/or western blotting. The current study showed that IL-13 increased the expression levels of p-mTOR, p-S6K1, and p-Akt, and that rapamycin blocked IL-13-induced down-regulation of miR-143, suppressed the IL-13Rα1 expression and up-regulated the expressions of filaggrin, loricrin, and involucrin in HaCaT cells. This study proposed that IL-13 could activate the mTOR signaling pathway, and confirmed the vital role of mTOR-miR-143 signaling axis in the pathogenesis of AD. It provided solid evidences regarding rapamycin as a potential effective therapeutic option in the management of AD.

## Introduction

Atopic dermatitis (AD) is a common chronic inflammatory dermatosis affecting up to 20% of different populations worldwide 1. It occurs mainly in children, with some of the cases persisting into adulthood. Clinically, it usually presents as recurring eczematous lesions with intense itching. AD is usually associated with personal or family history of atopic diseases, including food allergies, allergic rhinitis, and asthma, as well as cardiovascular diseases 2,3. AD presents a significant therapeutic challenge. Patients with AD suffer from a remarkably impaired quality of life.

The pathogenesis of AD mainly contains the dysfunction of epidermal barrier structure and the immune system 4,5. The skin barrier function repair remains the mainstay in the management of AD. Type2 T helper (Th2) cells are considered to play an essential role in the immune abnormalities of AD 6,7. Interleukin (IL)-13, a Th2 cytokine, is suggested to suppress the expression of filaggrin (FLG), loricrin (LOR), and involucrin (IVL) in keratinocytes 4,8, which are important proteins for skin barrier structure. The IL-13 signaling pathway is believed to be regulated through microRNAs (miRNAs) 9-11. However, evidences regarding the role of miRNAs in AD are limited.

Our preliminary study has shown that forced expression of microRNA-143 (miR-143) could block IL-13-induced down-regulation of epidermal barrier related proteins in keratinocytes of AD patients through directly targeting IL-13 receptor α1 (IL-13Rα1) 12. Emerging evidence suggested that the expression of miR-143 was regulated by mammalian target of rapamycin (mTOR) signaling pathway 13, which was identified as an essential regulator in epidermal morphogenesis 14. Naeem et al. 15 have highlighted the role of mTORC1-AKT1-CTSH axis in filaggrin expression in patients with AD. Furthermore, Hua et al. 16 found that mTOR inhibitor rapamycin inhibited eosinophil differentiation and significantly attenuated allergic airway inflammation in mice. Mushaben et al. 17 suggested that rapamycin could block increases in IgE and airway hyperreactivity, probably due to the reduction of IL-13 and leukotrienes in allergic asthma. However, the underlying molecular mechanism remains unclear.

In this study, we explored the effects of rapamycin on IL-13-stimulated keratinocytes, and investigated the molecular mechanism of mTOR-miR-143 signaling axis in the regulation of epidermal barrier related proteins. This study may give insight into the underlying mechanism of epidermal barrier dysfunction in AD, and provide essential theoretical basis for the clinical application of mTOR inhibitors in the management of AD.

## Materials and Methods

### Cell culture and treatment

HaCaT cells, which are immortal human keratinocytes, were cultured in Dulbecco's modified eagle medium with 1.3 mmol/L calcium (PromoCell GmbH, Heidelberg, Germany) at 37 °C, 5% CO_2_. 24h prior to treatment, HaCaT cells were seeded at 2×10^5^ cells/mL. Then, HaCaT cells were treated with 50 ng/mL of recombinant human IL-13 cytokine (R&D systems, Minneapolis, MN) for 48 h, except the blank control. 24 h after the stimulation, one group of HaCaT cells was treated with 20 ng/mL rapamycin (R&D systems), while another group with DMSO as the negative control.

### Quantitative real-time PCR (RT-qPCR)

Total RNAs were isolated using Trizol reagent (Invitrogen), assessed for the purity and concentration spectrophotometrically, and then reversely transcribed into cDNA with Revert Aid First Strand cDNA Synthesis Kit (Thermo Fisher Scientific Inc, MA, USA). RT-qPCR analysis were carried out in a 20 μL reaction system consisting of 5 μL cDNA template, 1 μL forward and reverse primers, and 10 μL SYBR Green qPCR Mix (Dong-sheng Bio, Guangzhou, China) on a Bio-Rad iQ5 RT-PCR System (Bio-Rad Laboratories, CA, USA). The PCR assays were performed as follows: 95°C for 2min, followed by 40 cycles of 95 °C 15 s and 60 °C for 20s, and subsequently 72 °C for 20 s. β-actin was used as an endogenous control. Forward and reverse primers against FLG, LOR, IVL, and IL-13Rα1 for PCR amplification were designed based on their sequences in the GenBank, consistent with our preliminary study 12.

Also, the reverse transcription of miRNA into cDNA and RT-qPCR analysis was performed using All-in-One miRNA qRT-PCR Detection Kit (GeneCopoeia, MD, USA). The PCR process was 95 °C for 10 min, followed by 40 cycles of 95 °C for 15 s and 60 °C for 20 s, and subsequently 72 °C for 20 s. A human U6 snRNAs was used as the endogenous control. Primers for the amplification of the cDNAs of hsa-miR-143a and hsa-U6 were purchased from GeneCopoeia.

### Western blotting

Total protein was extracted from HaCaT cells and protein concentration was determined using Bradford protein assay kit (Bio-Rad Laboratories). 20 μg of protein extracts were separated by 10% SDS-PAGE and electrotransferred onto a polyvinyllidenedifluoride membrane. The membranes were incubated overnight at 4 °C with primary antibodies against IL-13Rα1 (1:1000; Abcam, Cambridge, UK), p-S6K1 (Thr389, 1:1000, Cell Signaling Technology, USA), S6K1 (1:1000, Cell Signaling Technology, USA), p-Akt (Ser473, 1:2000, Cell Signaling Technology, USA), Akt (1:1000; Abcam, Cambridge, UK), p-mTOR (Ser2448, 1:1000, Cell Signaling Technology, USA), mTOR (1:1000, Cell Signaling Technology, USA), filaggrin (1:1000; Abcam, Cambridge, UK), loricrin (1:200; Santa Cruz Biotechnologies, CA, USA), involucrin (1:1000; Abcam, Cambridge, MA, USA), and β-actin (1:1000; Abcam, Cambridge, UK). The secondary antibodies (Sigma-Aldrich, St. Louis, MO) were incubated in 5% skimmed milk powder. The SuperSignal West Pico Chemiluminescent Substrate (Thermo Fisher Scientific Inc) was used to visualize the signals.

### Statistical analysis

All experiments were done in triplicate. Data were analyzed using the SPSS22.0 software and expressed as mean ± standard error of mean (SEM). Statistical significance was analyzed by two-tailed unpaired Student's *t* test and *p*<0.05 was considered statistically significant.

## Results

### Rapamycin blocked IL-13-induced down-regulation of miR-143

**Figure 1:**
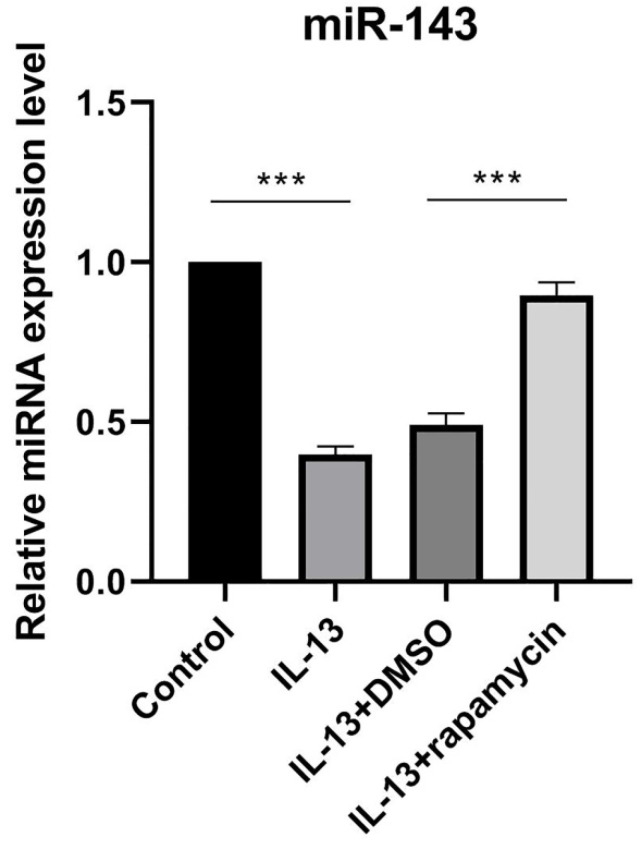
miR-143 level was decreased in IL-13-stimulated HaCaT cells, and markedly increased after treatment with rapamycin. All data are presented as the mean ± SEM of four different groups (***p<0.001, n = 3).

We previously reported that IL-13 inhibited miR-143 expression in cultured human epidermal keratinocytes 12. Additionally, previous studies demonstrated that mTOR inhibitor rapamycin could significantly up-regulate the expression of miR-143 13. In order to verify the effects of rapamycin on miR-143 expression in HaCaT cells, miR-143 levels were determined by RT-qPCR in different HaCaT cell groups (Fig. 1). The miR-143 level was decreased in IL-13-stimulated HaCaT cells, which was reversed by rapamycin treatment (Fig. 1).

### Down-regulation of IL-13Rα1 by rapamycin

IL-13Rα1 has been reported as a direct target and inhibited by miR-143 in human mast cells and nasal epithelial cells 11,18. Moreover, our preliminary study also showed that miR-143 targeted and decreased the production of IL-13Rα1 in primary normal human epidermal keratinocytes. In order to explore whether rapamycin could regulate the expression of IL-13Rα1 in HaCaT cells, we measured the mRNA and protein levels of IL-13Rα1 in IL-13-stimulated HaCaT cells through RT-qPCR and western blotting.

**Figure 2:**
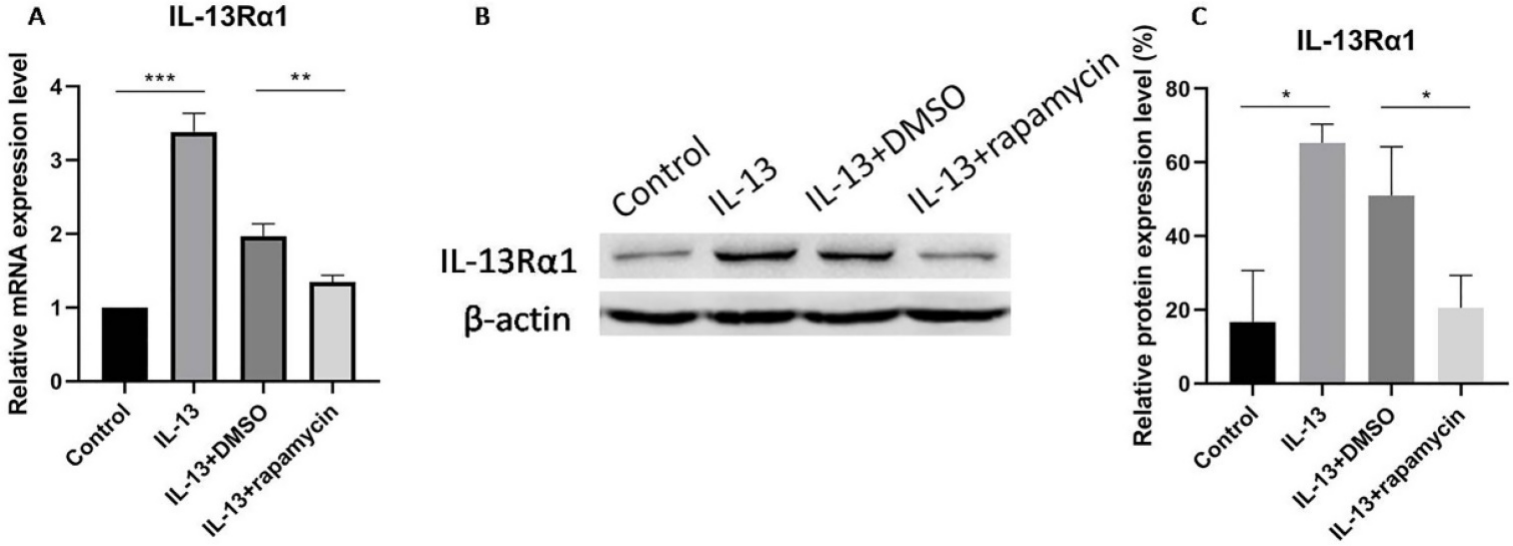
IL-13Rα1 was significantly suppressed at mRNA (**A**) and protein levels (**B,C**) after treatment with rapamycin. All data are presented as the mean ± SEM of four different groups (*p<0.05, **p<0.01, ***p<0.001, n = 3).

IL-13Rα1 was up-regulated in IL-13-stimulated cells which was consistent with our preliminary study. The expression of IL-13Rα1 was significantly suppressed at both mRNA (Fig. 2A) and protein (Fig. 2B, C) levels after treatment with rapamycin, compared with DMSO group.

### Activation of mTOR by IL-13 in HaCaT cells

**Figure 3:**
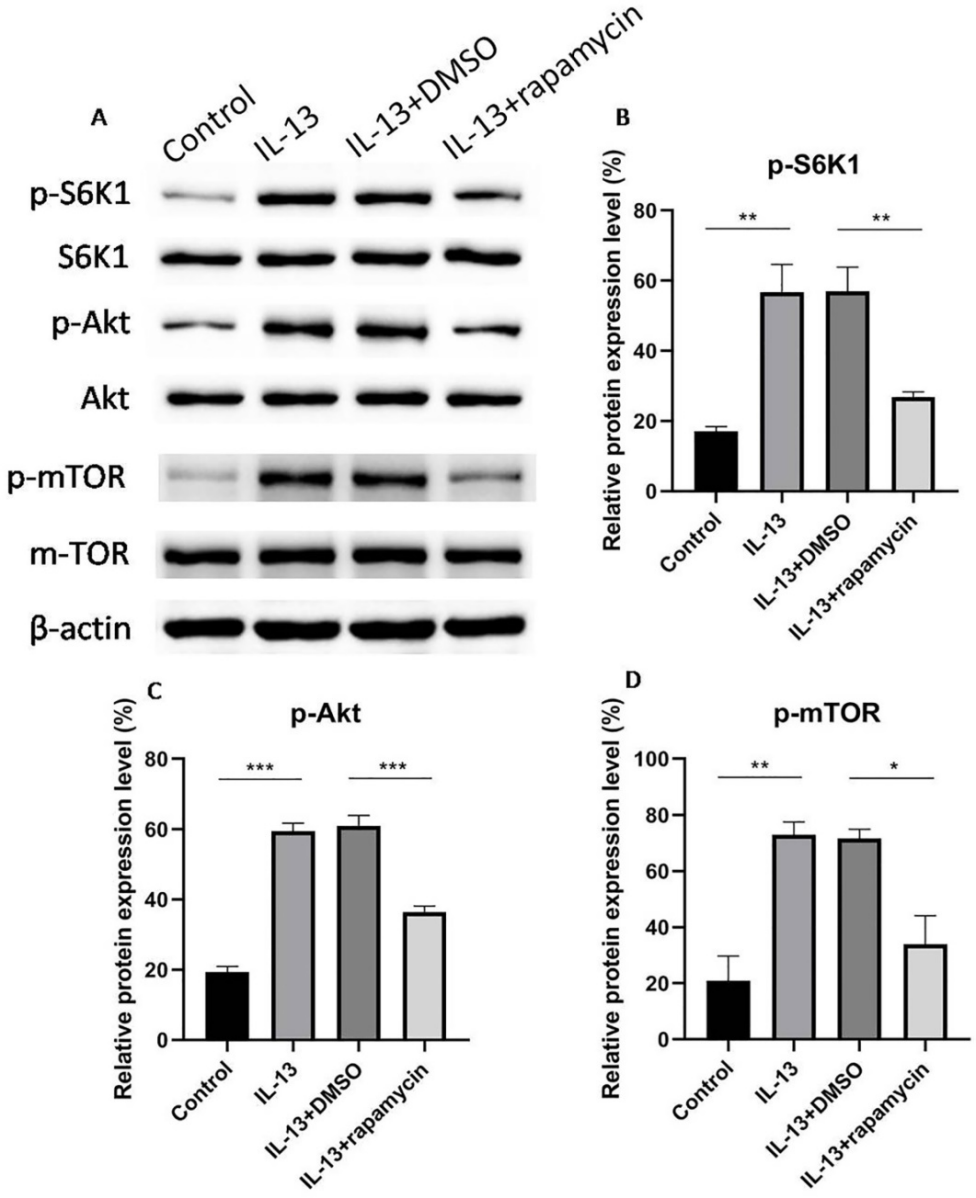
Western blotting analyses of phosphorylated S6K1, Akt, and mTOR (**A**). S6K1 (**B**), Akt (**C**) and mTOR (**D**) were activated by IL-13, and then inhibited significantly by rapamycin in HaCaT cells. All data are presented as the mean ± SEM of four different groups (*p<0.05, **p<0.01, ***p<0.001, n = 3).

mTOR is composed of two complexes, mTORC1 and mTORC2, which mediate the phosphorylation of S6K1 and Akt, respectively. In order to verify whether mTOR was involved in the regulation process, phosphorylated mTOR (p-mTOR), phosphorylated S6K1 (p-S6K1), and phosphorylated Akt (p-Akt) were assessed for the activation of mTOR using western blotting. mTOR, S6K1, and Akt were activated by IL-13, while the activation was inhibited by rapamycin (Fig. 3A-D).

### Up-regulation of FLG, LOR, and IVL by rapamycin

We previously observed that the overexpression of miR-143 could increase FLG, LOR, and IVL at mRNA level and markedly promote the synthesis of FLG, LOR, and IVL proteins. As the above results showed, miR-143 expression was up-regulated by rapamycin. To elucidate the effect of rapamycin on the regulation of these proteins, the mRNA and protein levels of FLG, LOR, and IVL were analyzed using RT-qPCR and western blotting.

**Figure 4:**
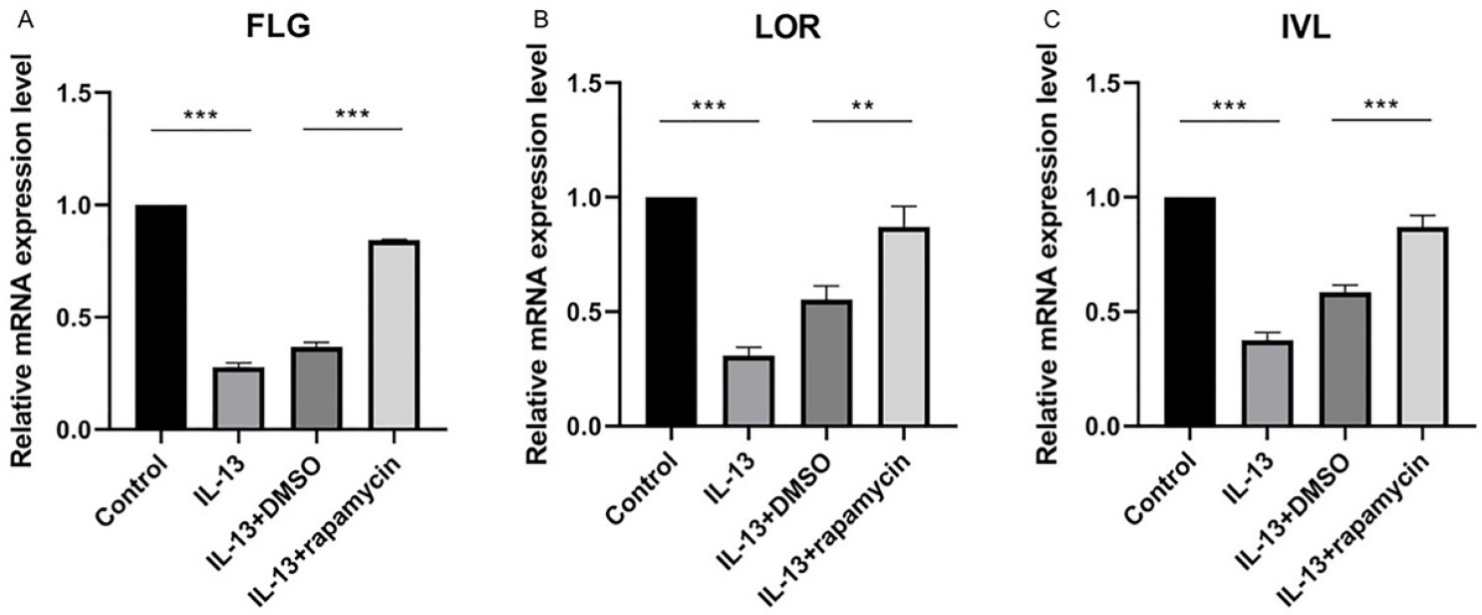
The relative mRNA levels of FLG (**A**), LOR (**B**), and IVL (**C**) were significantly up-regulated after treatment with rapamycin. All data are presented as the mean ± SEM of four different groups (**p<0.01, ***p<0.001, n = 3).

**Figure 5:**
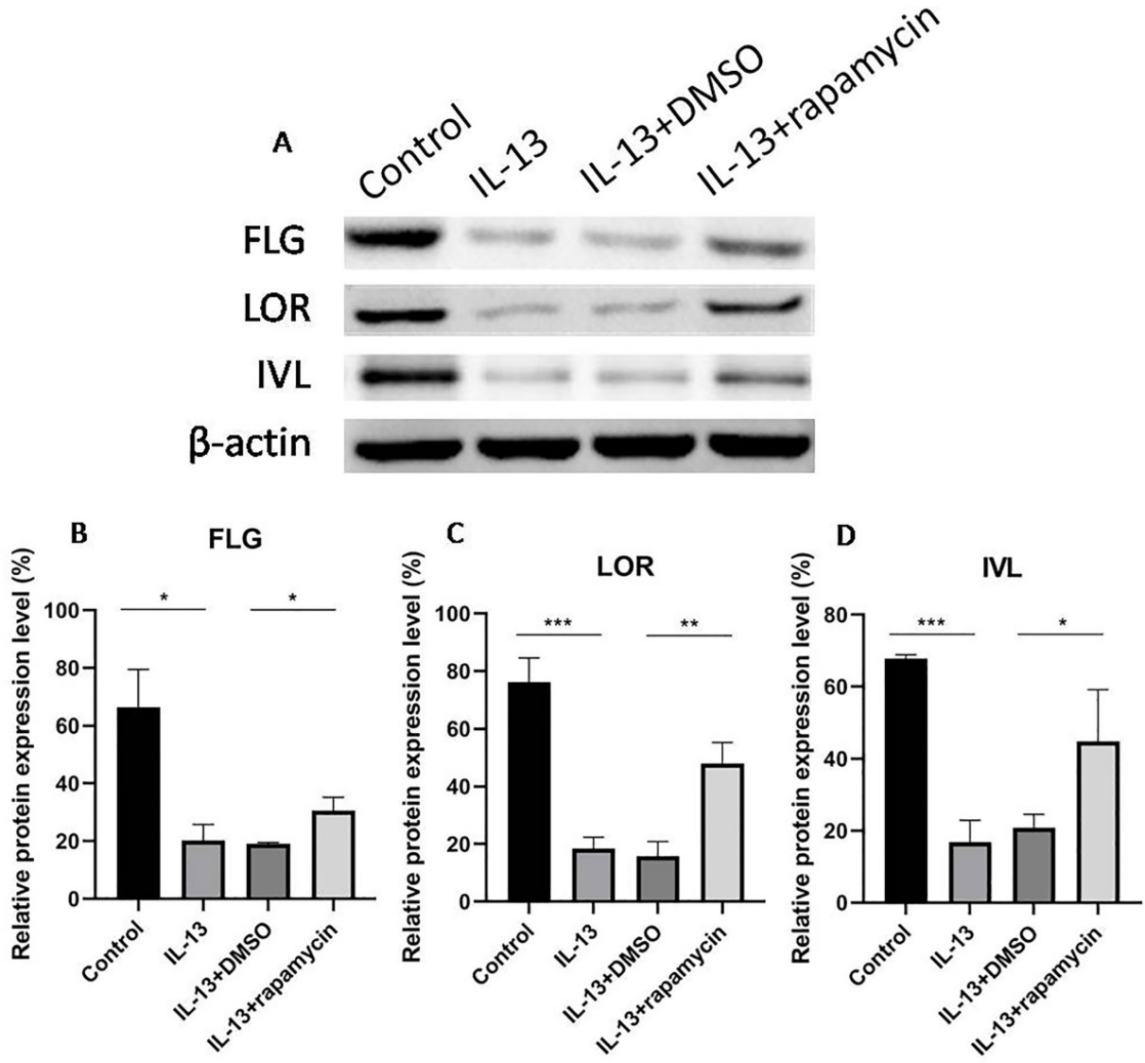
Western blotting analyses of FLG, LOR, and IVL expression (**A**). The expressions of FLG (**B**), LOR (**C**), and IVL (**D**) were suppressed by IL-13, and significantly up-regulated at protein levels after treatment with rapamycin. All data are presented as the mean ± SEM of four different groups (*p<0.05, **p<0.01, ***p<0.001, n = 3).

The expressions of FLG, LOR, and IVL were suppressed by IL-13 and significantly up-regulated at mRNA (Fig. 4A-C) and protein levels (Fig. 5A-D) after treatment with rapamycin.

## Discussion

AD is widely regarded as a joint effect of genetic and environmental factors, dysfunction of immune system and epidermal barrier structure. AD is characterized by dermal inflammatory cell infiltration, mainly with Th2 cells 4. Th2 cytokines have been proved to be overexpressed in the lesions of acute AD patients, especially IL-13 19.Consistent with earlier researches, our study revealed that IL-13 inhibited the mRNA and protein expression of FLG, LOR, and IVL in HaCaT cells, and supported the essential role of IL-13 in the immune dysfunction and abnormal barrier homoeostasis of AD.

miRNAs are believed as key regulators in allergic diseases (asthma and allergic rhinitis) and cutaneous inflammatory disorders (psoriasis) 20,21. miRNAs are a group of single-stranded, non-coding, short RNAs with the length of approximately 22 nucleotides. Through binding to the 3' untranslated regions of mRNA transcripts, miRNAs could inhibit or promote translation, or induce the degradation of target mRNAs, and consequently regulate gene expression and alter biological function 22,23. Numerous studies have found that miR-143, located at 5q33, was down-regulated in a variety of epithelial malignancies, including colon, pancreatic, and gastric cancers. miR-143 has been observed to block tumorigenesis and deemed as a tumor suppressor 24-26. Recently, a miRNA microarray assay by Yu et al. 27 revealed that miR-143 was the most remarkably down-regulated miRNA in nasal mucosa tissues with allergic rhinitis. Teng et al. 11 found that overexpression of miR-143 decreased the expression level of IL-13-induced inflammatory cytokines and mucus production in nasal epithelial cells from allergic rhinitis patients.

Consistent with our preliminary study, the current study confirmed that IL-13 inhibited miR-143 expression in cultured HaCaT cells. Moreover, we have already demonstrated that miR-143 promoted the expression of epidermal barrier-related proteins which were previously down-regulated by IL-13, and intimated that miR-143 may be regarded as potential therapeutic target in AD 12. However, the detailed mechanism of miR-143 in the pathogenesis of AD remains unknown.

In this study, we demonstrated that the miR-143 level was significantly increased after the blockade of mTOR with rapamycin in IL-13-stimulated HaCaT cells. This result was consistent with the result of an earlier study conducted by Fang et al. 13 which demonstrated that mTOR inhibitors, rapamycin and PP242 could significantly up-regulate the expression of miR-143. It is generally known that the proliferation and metabolism of T cells are mediated via mTOR pathway in the differentiation process 28. The activation of mTOR could promote T cell differentiation towards T helper cells, including Th2 cells 29. Moreover, Salmond et al. 30 found that IL-13 production of Th2 cells were reduced due to the inhibition of mTOR activity. Similarly, Hua et al. 16 confirmed that rapamycin could reduce the production of IL-13. Therefore, reduced activity of mTOR may decrease the expression of IL-13 and lead to the up-regulation of miR-143, which explains the effect of rapamycin on the miR-143 expression observed in this study.

We previously found that IL-13Rα1 expression was down-regulated by miR-143. In this study, we shown that, IL-13Rα1 was negatively regulated at both mRNA and protein levels after treatment with rapamycin. Moreover, in a study of allergic rhinitis, miR-143 was confirmed to inhibit IL13Rα1 gene expression and probably resulting in the suppression of IL-13-induced inflammatory cytokine and mucus 11. Based on the above results, we suggested that IL-13 activity was regulated through mTOR-miR-143 signaling axis by targeting IL-13Rα1.

Intriguingly, western blotting analysis revealed that the expression of p-mTOR, p-S6K1, and p-Akt were apparently increased in the cell group treated with IL-13, compared to the control group, indicating that IL-13 activated mTOR signaling pathway in HaCaT cells. Dysregulation of mTOR signaling pathway is proved to be involved in a variety of pathological process, including cancer, diabetes and neurodegeneration 28. Recent investigations demonstrated that abnormal mTOR activity was associated with the pathogenesis of inflammatory rheumatic diseases and autoimmune diseases, including rheumatoid arthritis, systemic lupus erythematosus and systemic sclerosis 31,32. Our results suggested that mTOR signaling pathway could be activated by IL-13 and play a major role in the pathogenesis of AD.

Rapamycin is considered with broad function of anti-proliferation and immunosuppression. As expected, rapamycin blocked the down-regulation of FLG, LOR, and IVL induced by IL-13 in our study, indicating that rapamycin could block epidermal barrier dysfunction and maintain abnormal permeability barrier homoeostasis. Jung et al. 33 have also demonstrated that topical 4% rapamycin treatment significantly improved clinical symptoms including erythema, edema, dryness and excoriation in AD mice, through inhibiting the expression of IL-4 and IFN-γ. Therefore, rapamycin might provide a potential therapeutic option in the management of AD.

Additionally, we found that the activation of both S6K1 and Akt were blocked by rapamycin. Recent studies have reported that rapamycin could block the phosphorylation of S6K1 and Akt in neuronal cells, and attenuate apoptotic cell death 34. Moreover, Zeng et al. 35 showed that rapamycin inhibited phosphorylation of S6K1 and Akt in B cells, indicating that rapamycin prevented B cell proliferation through both mTORC1 and mTORC2 pathways. Our study demonstrated that rapamycin blocked the IL-13-induced deficiency of epidermal barrier related proteins not only via targeting mTORC1-mediated S6K1 pathway, but also through mTORC2-mediated Akt pathway.

**Figure 6:**
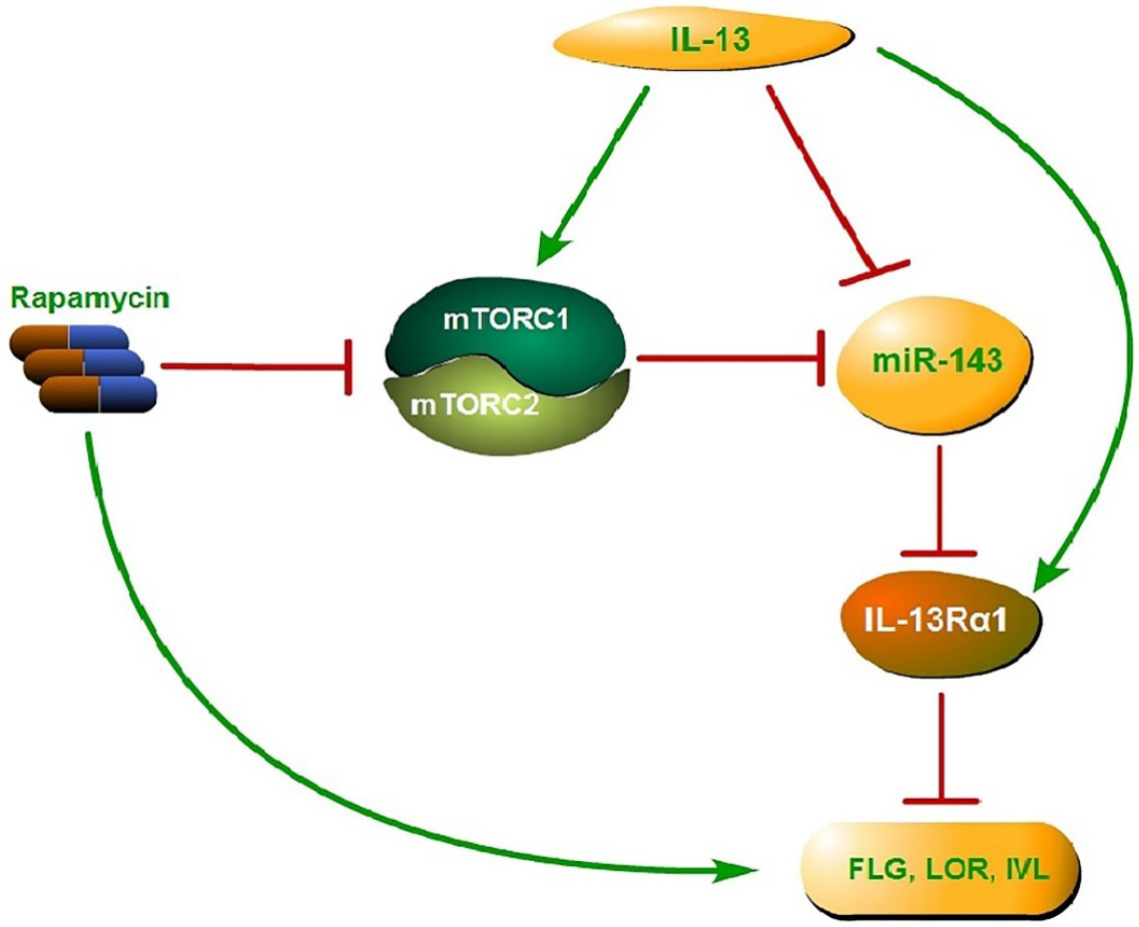
Proposed mechanism of mTOR-miR-143 signaling axis in the pathogenesis of AD. Activated mTOR signaling pathway by IL-13 could down-regulate the miR-143 expression and then block the down-regulation of IL-13Rα1 by miR-143, which results in the enhancement of IL-13 activity and suppression of epidermal barrier-related proteins. The above process could be reversed by rapamycin, which targets both mTORC1 and mTORC2, leading to up-regulation of epidermal barrier-related proteins.

Collectively, activated mTOR signaling pathway by IL-13 could down-regulate the miR-143 expression and then block the down-regulation of IL-13Rα1 by miR-143, which results in the enhancement of IL-13 activity and suppression of epidermal barrier-related proteins. The above process could be reversed by rapamycin, which targets mTOR, leading to up-regulation of epidermal barrier-related proteins (Fig. 6). In conclusion, the present study confirmed the vital role of mTOR-miR-143 signaling axis in the pathogenesis of AD, and provided solid evidences regarding rapamycin as a potential effective modality in the treatment of AD.

## Supplementary Material

Supplementary figure.
