# Supplementary material for: Rapamycin blocks the IL-13-induced deficiency of Epidermal Barrier Related Proteins via upregulation of miR-143 in HaCaT Keratinocytes
Source: Int J Med Sci. 2020 Jul 25;17(14):2087–94. doi: 10.7150/ijms.45765 (PMC7484670; doi:10.7150/ijms.45765)
Supplement: Supplementary file 1 — Supplementary figure. [file ijmsv17p2087s1.pdf]

Supplementary figure 1.

Semi-quantitative analyses of the protein expression of S6K1 (A), Akt (B), and mTOR (C) between the control group and IL-13 treated group or between the IL-13+DMSO treated group and IL-13+rapamycin treated group showed no significant differences. *ns*= no significant differences.

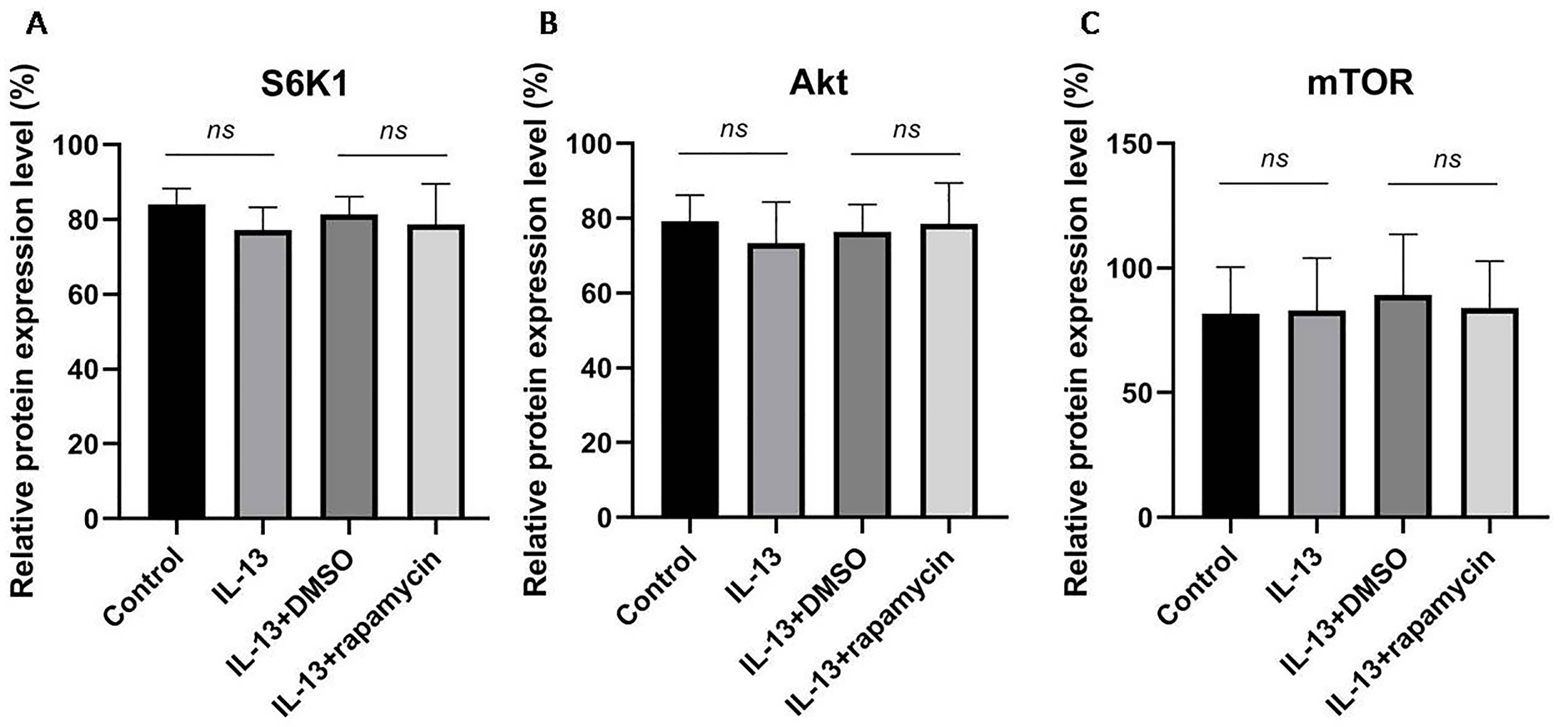
A
S6K1
Relative protein expression level (%)
100
80
60
40
20
0
ns
ns
Control
IL-13
IL-13+DMSO
IL-13+rapamycin
B
Akt
Relative protein expression level (%)
100
80
60
40
20
0
ns
ns
Control
IL-13
IL-13+DMSO
IL-13+rapamycin
C
mTOR
Relative protein expression level (%)
150
100
50
0
ns
ns
Control
IL-13
IL-13+DMSO
IL-13+rapamycin
